# Wear Behavior and Water Sorption of Additively Manufactured Resin-Based Splint Materials

**DOI:** 10.3390/ma17235880

**Published:** 2024-11-30

**Authors:** Johann Wulff, Cordula Leonie Merle, Sebastian Hahnel, Martin Rosentritt

**Affiliations:** Department of Prosthetic Dentistry, UKR University Hospital Regensburg, 93042 Regensburg, Germany; johann-philip.wulff@ukr.de (J.W.);

**Keywords:** 3D printing, additive manufacturing, DLP, oral splint, TMD, water sorption, wear behavior

## Abstract

3D printing as an additive manufacturing method has proven to be of great interest for the computerized production of oral splints. Various parameters must be taken into consideration when assessing the durability of oral splints in a wet environment, such as the mouth. The aim of this in vitro study was to assess the wear behavior and water sorption of two 3D-printed splint materials depending on their building orientation and post-processing parameters. The parameters considered included the type of post-polymerization and the type of cleaning utilized after printing. The average wear depth was between −421.8 μm and −667.5 μm. A significant influence of the building orientation (*p* < 0.001) but not of the material (*p* = 0.810), cleaning (*p* = 0.933), or post-polymerization (*p* = 0.237) on wear was demonstrated. The water sorption ranged between 13.8 μg/mm^3^ and 30.3 μg/mm^3^, featuring a significant dependency on material and building orientation but not on cleaning (*p* = 0.826) or post-polymerization (*p* = 0.343). Material and fabrication methods should be carefully selected, because the type of material and building orientation affect the wear and water sorption of additively manufactured splint materials.

## 1. Introduction

Patients in dental practices around the world have tooth wear. A certain amount of tooth wear is regarded physiological, but in some patients, wear is higher and can be classified as pathological [1]. Changes in tooth structure can be caused by a normal exposure to life as a result of eating or chewing, but also by bruxism [2]. Bruxism may manifest itself during the day or during the night as awake bruxism or night bruxism, respectively [3]. Recent studies have reviewed the adverse consequences of bruxism on the stomatognathic system [4]. Nevertheless, there is evidence that a certain degree of bruxism-related activity of the masticatory muscles is not necessarily pathological. [5]. Well-established therapeutic options include those implied in the “multiple-P” approach, i.e., plates, pep talk, and pills [3,6,7]. Oral splint therapy, i.e., plates, is regarded as an evidence-based therapeutic option for the management of many temporomandibular disorders (TMD) and parafunctions of the stomatognathic system, such as bruxism in its various presentations [8,9]. Oral splints can be manufactured either conventionally, digitally, or with a hybrid approach. The efficacy of the conventional approach has been established in recent decades in dentistry. Conventional methods of splint production include cast methacrylates, deep drawn thermoplastics, vacuum injection molding, scattering of acrylic resin, or a combination of the latter [10,11,12]. The analogous workflow normally consists of taking impressions of the patients’ upper and lower arches, cast fabrication, wax-up, and splint production in the aforementioned manners. Commonly, the dimensional changes in the impression materials and the cast used to fabricate the models lead to a higher risk of inferior accuracy in the fit of oral splints. The quality and clinical performance of analogously fabricated oral splints are affected by the residual monomer content and shrinkage, which occur during the production process [13]. 

Recently, though, advancements in digital technology have played an important role regarding the production of dental appliances. Following computerized optical scans of the maxilla and mandible and digital bite registration, dental appliances are likewise designed in a digital manner. Computer-aided design (CAD) and computer-aided manufacturing (CAM) are now commonly used by dental professionals in their daily practice [14]. Digital workflows have been proven to be both cost-effective and time-efficient, facilitating the production of precise and reproducible results [15]. Many dental laboratories use subtractive milling units as part of the digital workflow in order to produce oral splints. Nevertheless, in comparison with their milled counterparts, splints manufactured by additive manufacturing offer economic and ecological benefits [16]. While milling is regularly carried out utilizing industrially manufactured PMMA blanks, digital light processing is carried out using liquid photopolymer resins. Regarding milling, one prepolymerized blank is sufficient for milling of one to two splints, which, however, results in a large amount of non-reusable waste [17,18]. Producing splints with additive manufacturing methods may be more sustainable. However, recent studies have noted that subtractively and conventionally manufactured splints show better mechanical properties than additively manufactured ones [15,19,20,21]. Nonetheless, additively and subtractively produced splints seem to have mechanically and chemically acceptable properties for clinical use [22]. 

As previously mentioned, the mechanical properties of additively manufactured splint materials play a crucial role in selecting the appropriate material for splint production. Chemical and mechanical properties influence the clinical performance of the appliances. A splint with reduced wear resistance and subsequently reduced flexural strength might show reduced service life [22]. Therefore, the wear resistance of splint materials has been a topic of interest for the last couple of decades. Wear can be defined as the progressive and cumulative degradation of a surface material or its deformation resulting from the mechanical contact between two moving surfaces [23]. Measuring wear parameters by determining the volumetric loss seems to be the most efficient way to quantify the change in the samples’ surface. It is a function of depth and area and can be translated into clinical wear [23]. 

Printed splints feature a higher level of flexibility than their hand-cast or milled counterparts yet maintain an adequate level of strength. Many studies have postulated that the thickness and orientation of the layers during the printing process have an influence on the mechanical properties of the splints and, therefore, their clinical performance [24,25,26,27]. However, other parameters should also be taken into consideration when evaluating the performance of oral splints. The exposure of materials to a humid or wet environment normally leads to degradation of their mechanical properties. This phenomenon could support the assumption that reduced water sorption in clinical settings leads to better long-term stability in the oral cavity [28,29]. Accelerated aging is often used to describe the long-term wear behavior of composites. Crack initiation and expansion in composites can be caused by an increased diffusion rate of water molecules in the resin matrix through elevated temperatures and humidity [30]. Different models have been proposed to characterize the water sorption and diffusion behavior of composites. One of these is the Fick diffusion model, which assumes that the material is homogenous and the diffusion takes place under the influence of the concentration gradient. Alternatively, the stress-dependent diffusion model (Langmuir) and the two-stage diffusion model have been proposed to explain non-Fick diffusion behavior in composite materials [31]. Water molecule penetration and diffusion in composites are associated with the molecular chain structure in resin-based materials. The understanding and investigation of water sorption of resin-based materials seems to be of great importance in order to explain wear behavior and service life of resin-based restorations [30]. 

Given the increasing prevalence of digital processes in dental offices, such as dental milling devices and 3D printers, it is crucial to provide sound guidance on material selection and processing. 

It was, therefore, the objective of this in vitro study to assess the wear behavior and water sorption of two different additively manufactured splint materials while considering the influence of the pre- and post-processing parameters. The results were analyzed and summarized in order to provide guidance when selecting and processing splint materials for additive manufacturing. The hypothesis of this research was that the wear behavior and water sorption of the utilized resin-based splint materials are affected by the building orientation of the samples to the building platform, manual or automated cleaning, LED- or Xenon post-polymerization, or storage of the tested samples. 

## 2. Materials and Methods

### 2.1. Printing—Wear Behavior/Water Sorption

**Figure 1:**
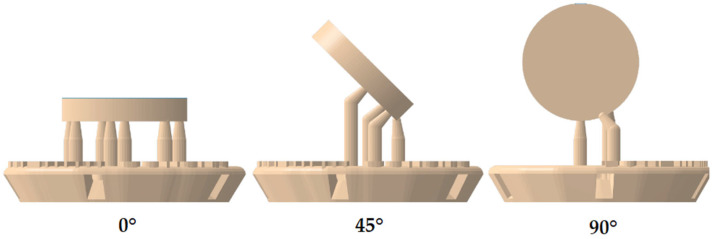
Sample design—orientation to building platform.

Samples for both trials were printed from two different splint materials (M1: Luxaprint OrthoPlus, DMG, Hamburg, Germany; M2: V-Print Splint, VOCO, Cuxhaven, Germany) were printed utilizing a P30+ DLP printer (Straumann, Basel, Switzerland) (Table 1). The printed samples had a diameter of 10 mm and a height of 2 mm. The three-dimensional printing process was performed positioning the samples at 90 degrees, 45 degrees, or 0 degree angles to the building platform (Figure 1). The samples were designed with supporting structures, and the thickness of the layers was 100 μm. A total of 504 samples were printed. Wear behavior was tested in 384 of these samples, while 120 samples were used to test water sorption. 

### 2.2. Cleaning—Wear Behavior/Water Sorption

After completing the printing process, the samples were subjected to cleaning by utilizing either an automated (AUTO: P Wash, Straumann, Basel, Switzerland) or a manual (MAN: VOCO Pre-/Main-Clean protocol, VOCO, Cuxhaven, Germany) cleaning procedure. Isopropanol was utilized as a cleaning agent in each of the cleaning protocols. 

### 2.3. Post-Polymerization—Wear Behavior/Water Sorption

After the samples were cleaned, these were subjected to a post-polymerization process. External curing appliances using either LED (LED: P Cure, Straumann, Basel, Switzerland) or Xenon lights (XEN: Otoflash N171, Ernst Hinrichs Dental, Goslar, Germany) were utilized for this part of the process. 

### 2.4. Processing

#### 2.4.1. Processing—Wear Behavior

The printed samples were processed and tested according to ISO/TR 14569-1:2007-05 [32]. Samples were ground to the final dimensions (diameter 10 mm, height 2 mm) by utilizing silicon carbide paper (P1200/4000; Struers GmbH, Willich, Germany), pumice stone powder, and universal polishing paste on the surface to be tested (Ivoclar Vivadent AG, Schaan, Lichtenstein). 

#### 2.4.2. Processing—Water Sorption 

The printed samples were processed and tested according to DIN EN ISO 10477:2021-02 [33]. Samples were ground on all sides to the final dimensions (diameter 10 mm, height 2 mm) by using silicon carbide paper (P1200/4000; Struers GmbH, Willich, Germany), pumice stone powder, and universal polishing paste (Ivoclar Vivadent AG, Schaan, Lichtenstein). All samples were cleaned in an ultrasonic bath for 2 min to remove any excess material, rinsed with distilled water, and dried with compressed air.

### 2.5. Storage

#### 2.5.1. Storage—Wear Behavior

After processing, samples were stored in distilled water at 37 °C for either 24 h (n = 192) or 60 days (n = 192).

#### 2.5.2. Storage—Water Sorption 

The storage of the samples is elaborated on in another Section (2.6.2. Testing—Water sorption and solubility) since it is a part of the testing process. 

### 2.6. Testing

#### 2.6.1. Testing—Wear Behavior

The samples were subjected to dynamic loading in a dual-axis mastication simulator (EGO; Goldbach GmbH, Regensburg, Germany). A load of 50 N, lateral movement of 1 mm, and vertical movement of 1 mm were applied 40 000 times at a frequency of 1.2 Hz in a distilled water bath (21 °C). The load was applied with a steatite sphere (CeramTec GmbH, Plochingen, Germany) 3 mm in diameter onto the central part of the sample. Wear depth as well as Ra (average surface roughness) and Rz (maximum peak to valley height at 5 regions) in and next to the wear trail were determined using a laser microscope (VK-X105, Keyence, Osaka, Japan). 

#### 2.6.2. Testing—Water Sorption and Solubility 

The volume (*V*, in mm^3^) of each sample was calculated by averaging two diameter measurements and four equally spaced thickness measurements around the circumference. The samples were placed in racks inside desiccators (Duran; DWK Life Sciences GmbH, Wertheim, Germany), which contained freshly dried silica gel (Merck KGaA, Darmstadt, Germany), stored at 37 ± 1 °C for 23 ± 1 h, and weighed on an analytical balance, accurate to 0.0002 g (Sartorius AG, Goettingen, Germany). This drying and weighing cycle was repeated until a constant mass, *m*_1_, also known as ‘conditioned mass’, was reached. The conditioned mass was determined when the difference between two consecutive weight measurements was less than 0.1 mg. The samples were then stored in distilled water at 37 ± 1 °C. The samples were removed from water after seven days, wiped gently until free from visible moisture, and weighed 60 s after removal. This weight measurement, *m*_2_, was called ‘water saturation’. The samples were then again placed in the desiccator. The desiccation procedure described above was repeated until constant mass was reached, *m*_3_, called ‘reconditioned mass’. The water sorption, *W_sp_*, and water solubility, *W_sl_*, were expressed in μg/mm^3^ using the following equations:

*W_sp_* = (*m*_2_ − *m*_3_)/*V* (1)

*W_sl_* = (*m*_1_ − *m*_3_)/*V* (2)

Legend: 

*W_sp_* = water sorption (μg/mm^3^);

*W_sl_* = water solubility (μg/mm^3^);

*m*_1_ = constant mass dry samples (μg);

*m*_2_ = constant mass wet samples (μg);

*m*_3_ = constant mass reconditioned samples (μg);

*V* = volume of samples (mm^3^).

### 2.7. Statistics

Statistical analysis was performed with SPSS 28.0 (IBM, Armonk, NY, USA, α = 0.05). Means and standard deviations were calculated and analyzed using one-way analysis of variance (ANOVA) and the Bonferroni test for post hoc analysis. Between-subjects effects were investigated (Table 2 and Table 3). Pearson correlation between the individual parameters was determined. 

## 3. Results

### 3.1. Wear Behavior

#### 3.1.1. Building Orientation

**Figure 2:**
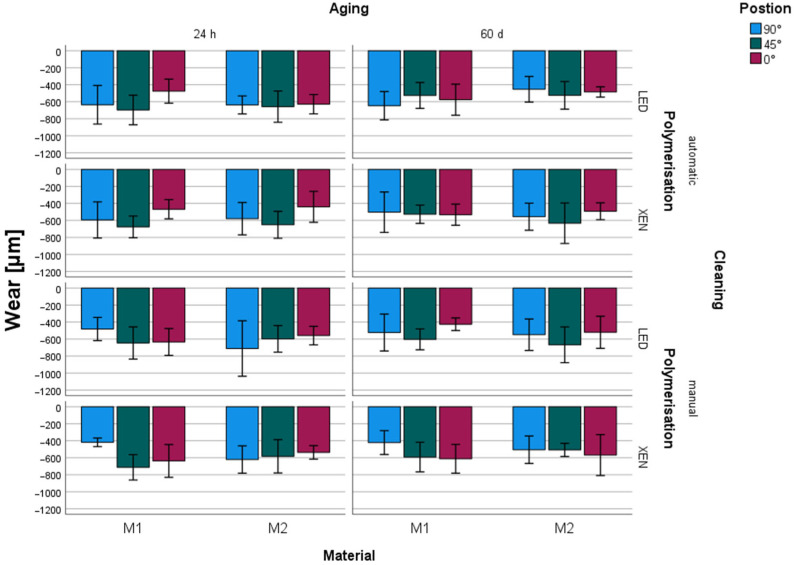
Wear (µm, mean standard deviation) depending on material, position, cleaning, and polymerization.

The mean wear of the tested samples varied between −418.6 µm and −711.3 µm for 90°, −507.9 µm and −713.0 µm for 45°, and −425.6 µm and −637.3 µm for 0° (Table 4, Figure 2). A significant influence of the building orientation on the wear behavior of the tested material could be observed (*p* < 0.001) (Table 2). 

#### 3.1.2. Cleaning

Depending on the cleaning procedure utilized, mean wear values ranged from −440.0 µm and −697.0 µm for AUTO and −418.6 µm and −713.0 µm for MAN (Table 4, Figure 2). No significant influence of the utilized cleaning procedure on wear behavior could be determined (*p* = 0.933). 

#### 3.1.3. Post-Polymerization

Regarding the light source for post-polymerization, mean wear values varied between −425.6 µm and −711.3 µm for LED and −418.6 µm and −713.0 µm for XEN (Table 4, Figure 2). No significant influence of the type of light source utilized could be observed (*p* = 0.237). 

#### 3.1.4. Storage

Mean wear after 24 h of storage varied between −418.6 µm (M1, 90°, MAN, XEN) and −713.0 µm (M1, 45°, MAN, XEN) and after 60 days between −421.8 µm (M1, 90°, MAN, XEN) and −667.5 µm (M2, 45°, MAN, LED), with significant differences (*p* = 0.002) between the results (Table 2, Table 4, Figure 2). 

A significant influence of storage (*p* = 0.001) and position (*p* = 0.001) could be observed. No significant influence could be determined for materials (*p* = 0.810), cleaning (*p* = 0.933), or polymerization (*p* = 0.237). Only between aging and wear a significant (Pearson: 0.158, *p* = 0.002) correlation was found (Table 2).

### 3.2. Water Sorption

#### 3.2.1. Building Orientation

**Figure 3:**
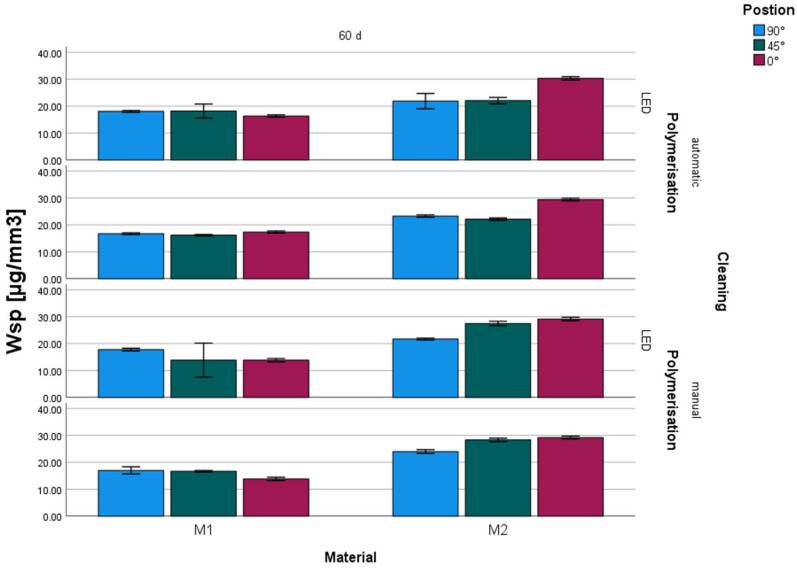
Water sorption Wsp (µg/mm^3^, mean standard deviation) depending on material, position, cleaning, and polymerization.

Regarding building orientation, the mean values for water sorption ranged between 16.7 µg/mm^3^ and 24.0 µg/mm^3^ for 90°, 13.8 µg/mm^3^ and 28.3 µg/mm^3^ for 45°, and 13.8 µg/mm^3^ and 30.3 µg/mm^3^ for 0° (Table 4, Figure 3). A significant influence of the building orientation could be observed (*p* < 0.001) (Table 3). 

#### 3.2.2. Cleaning

In terms of the utilized cleaning procedure, the mean values for water sorption ranged between 16.2 µg/mm^3^ and 30.3 µg/mm^3^ for AUTO and 13.8 µg/mm^3^ and 29.2 µg/mm^3^ for MAN (Table 4, Figure 3). No significant influence of cleaning on water sorption could be observed (Table 3). 

#### 3.2.3. Post-Polymerization

The mean water sorption after post-polymerization with LED light varied between 13.8 µg/mm^3^ and 30.3 µg/mm^3^. Post-polymerization with Xenon light produced values between 13.8 µg/mm^3^ and 29.4 µg/mm^3^ (Table 4, Figure 3). A significant influence of the post-polymerization method on water sorption of the tested materials could not be observed (Table 3). 

Water sorption varied between 13.8 µg/mm^3^ (M1, 0°, MAN, LED/XEN) and 30.3 µg/mm^3^ (M2, 0°, AUTO, LED) with significant differences (*p* < 0.001) between the results (Table 3 and Table 4). A significant influence of material (*p* < 0.001) and position (*p* < 0.001) could be observed. No significant influence could be determined for cleaning (*p* = 0.826) or polymerization (*p* = 0.343). Only between water sorption and material a significant (Pearson: 0.846, *p* < 0.001) correlation was identified (Table 3). 

**Table 4:** Wear [µm] and water sorption [µg/mm^3^] (mean standard deviation) depending on material, position, cleaning, and polymerization.

				Wear				Water Sorption	
				24 h		60 d			
Orientation to Building Platform	Material	Cleaning	Post- Polymerization	Mean [µm]	SD	Mean [µm]	SD	Mean [µm]	SD
90°	1	WA	LED	−635.5	227.1	−647.0	166.8	18.1	0.3
			XEN	−594.6	212.7	−503.4	237.9	16.7	0.3
		WM	LED	−481.6	136.5	−523.0	217.4	17.7	0.5
			XEN	−418.6	50.3	−421.8	140.4	17.0	1.3
	2	WA	LED	−637.6	105.6	−453.4	150.5	21.9	2.8
			XEN	−579.8	191.1	−556.5	159.0	23.3	0.4
		WM	LED	−711.3	326.1	−548.8	185.0	21.7	0.3
			XEN	−620.8	160.8	−506.1	161.1	24.0	0.7
45°	1	WA	LED	−697.0	174.0	−526.3	152.2	18.2	2.6
			XEN	−676.6	128.0	−527.5	107.7	16.2	0.3
		WM	LED	−645.9	189.4	−603.3	122.4	13.8	6.3
			XEN	−713.0	149.0	−592.2	174.1	16.7	0.3
	2	WA	LED	−657.5	184.3	−524.7	162.3	22.1	1.2
			XEN	−651.8	158.7	−633.1	238.3	22.1	0.5
		WM	LED	−597.5	156.5	−667.5	210.2	27.5	0.8
			XEN	−583.6	196.1	−507.9	77.1	28.3	0.7
0°	1	WA	LED	−476.0	141.1	−576.0	182.6	16.3	0.5
			XEN	−469.0	113.2	−533.1	124.4	17.3	0.4
		WM	LED	−634.1	158.4	−425.6	74.0	13.8	0.6
			XEN	−637.3	192.8	−612.2	169.4	13.8	0.6
	2	WA	LED	−628.9	113.8	−485.2	60.5	30.3	0.6
			XEN	−440.0	181.9	−492.2	99.6	29.4	0.5
		WM	LED	−559.0	109.1	−520.3	188.8	29.1	0.6
			XEN	−537.2	79.8	−568.6	240.8	29.2	0.5

## 4. Discussion

The hypotheses that wear behavior and water sorption of the tested additively manufactured splint materials are influenced by the building orientation of the objects in relation to the building platform (0°, 45°, 90°), the cleaning process after printing (MAN, AUTO), the post-polymerization in terms of the light source utilized (LED, XEN), and storage of the samples (24 h, 60 days—only wear behavior) could be partly confirmed.

Wear behavior was significantly influenced by the building orientation of the samples as well as by their storage in distilled water. Water sorption was significantly affected by the building orientation to the building platform as well as by the tested material. 

### 4.1. Wear Behavior

Wear behavior testing in this study was performed using a pin-on-disk approach. This method is widely utilized in order to examine the contact and sliding behavior of materials and important wear mechanisms under different tribological conditions [34]. Distilled water was used to simulate a wet environment, such as in the oral cavity. Using water as a lubricant instead of testing in a dry environment could lead to higher wear rates of polymers [35]. 

#### 4.1.1. Building Orientation

The wear behavior of the samples seemed to differ depending on whether they were printed at a 0°, 45°, or 90° angle to the platform (Figure 1). A significant influence of the parameters mentioned could be determined. The samples printed at an angle of 90° (M1, WM, XEN) showed the least wear after 24 h and 60 days of storage in distilled water. Samples printed at an angle of 45° (M1, WM, XEN) showed the highest wear after 24 h and 60 days of storage in distilled water (M2, WM, LED). Previous studies have shown that the flexural strength of materials decreased when a printing angle less than 90° in relation to the platform is used [24]. Other investigations have shown that the printing angle, layer thickness, and post-curing method also have an influence on the hardness of printed materials. Therefore, lower hardness values could be correlated to a decrease in wear resistance of the tested materials [17,29,36]. This observation is clinically relevant due to the fact that volumetric and vertical loss could change the static and dynamic relationship of the upper and lower jaws when using a splint [29]. Accordingly, earlier research has identified an anisotropy of additively manufactured materials, which was caused by the building orientation of the individual layers during printing [25,26,37,38]. 

#### 4.1.2. Cleaning

The wear behavior of the tested samples was also examined in relation to the cleaning technique used during processing. The samples were either manually or automatically cleaned as described above. The type of cleaning process did not seem to have a significant effect on the wear behavior of the samples in this study (Table 2). Previous studies have shown an influence of the cleaning process on some mechanical properties of additively manufactured materials, such as a decrease in flexural modulus dependent on cleaning time [39]. According to similar studies, flexural strength and biaxial flexural strength did not seem to decrease significantly in relation to the cleaning method utilized [24,39]. 

#### 4.1.3. Post-Polymerization

The type of light used for the post-polymerization did not seem to have an effect on the wear behavior of the tested materials (Table 2). Further studies have shown that the type of polymerization may affect some mechanical properties such as dynamic fatigue, biaxial flexural strength, hardness, and flexural strength [24,29,40,41]. The results in the present study would then lead to the assumption that both curing methods produced a sufficient degree of conversion (DC) on the outermost layers during post-polymerization. The fact that previous research determined that the layer thickness, the exposure duration, the distance to light source, and the curing time have an effect on other properties of the materials should not be overseen. DC and the material composition are of additional relevance in the medical field, as they play a crucial role in the biocompatibility of the materials [41,42,43]. Bayarsaikan et al. showed that an increase in post-polymerization temperature and time improved flexural properties, Vickers hardness, and biocompatibility of additively manufactured resin-based materials [44]. 

#### 4.1.4. Storage

A significant effect of the storage period could be observed as the wear resistance improved after storage (Table 2). Oh et al. investigated the effects of washing solution temperature on the biocompatibility and mechanical properties of additively manufactured dental resin materials [45]. A protocol of washing at 30 °C for 30 min presented a significantly improved biocompatibility of the materials without adversely impacting the bulk and surface mechanical behavior [45]. These results could lead to the conclusion that the degree of conversion of the tested samples was higher than in their counterparts in the 24 h group, which might be due to the increased temperature and exposure time during the 60-day storage period at 37 °C. 

### 4.2. Water Sorption

Additively manufactured resin-based devices are known to be more susceptible to water sorption and aging compared to their milled and pressed counterparts [8,46,47]. The nature of resin molecules and their polar properties are usually responsible for a higher tendency to absorb water or solvents over longer periods of time [48]. Barsby et al. observed that water sorption softens acrylic-based resins, leading to chemical degradation and monomer elution [49].

#### 4.2.1. Building Orientation

The anisotropy of additively manufactured materials has already been explained above. The orientation of the layers in the samples appears to be of great importance for water absorption (Table 3). The absorbed water penetrates into the layers and then diffuses into the polymer network, filling interstitial gaps with water and displacing the individual polymer chains [50,51]. This phenomenon may have negative effects on the interfaces, resulting in resin swelling and separation of printed layers [28]. The presence of these interstitial gaps and voids has been confirmed in previous research by scanning electron microscopy [50]. 

#### 4.2.2. Cleaning

A significant effect of the cleaning procedure could not be determined in this study (Table 3). Previous studies on temporary resins for additive manufacturing have shown a significant effect of washing agents and washing times on the mechanical properties. Liu et al. reported that alcohol-based solvents can lead to a deterioration of the mechanical properties. In contrast, the utilization of an organic solvent could produce improved mechanical properties and biocompatibility [52]. 

#### 4.2.3. Post-Polymerization

The type of curing process did not appear to have a significant effect on the water sorption of the materials. As explained above, post-polymerization can have a significant effect on the mechanical properties of printed materials (Section 4.1.3). However, previous studies on additively manufactured resins have shown that post-curing conditions did not affect the surface topography and roughness of the materials [53]. This observation, in turn, suggests that both post-curing methods led to a sufficient degree of transformation of the outermost layers of the tested materials.

#### 4.2.4. Material

Material composition varies from manufacturer to manufacturer and many producers do not specify the composition of their materials due to competitive reasons. However, the material behavior during and/or after processing may greatly vary depending on the individual composition of the tested resins. This study showed a significant difference in water sorption between the tested materials (Figure 2). 

Nevertheless, the current study has some limitations, e.g., as the results were obtained after testing two resin-based splint materials with a single DLP printer. To acquire a more comprehensive understanding of the available materials, future research could involve a larger number of products printed with more than one printer. Additionally, in vivo studies could be implemented in order to analyze the clinical performance of resin-based splint materials.

## 5. Conclusions

The present study investigated the effects of post-processing parameters on the wear behavior and water sorption of additively manufactured splint materials. Within the limitations of this research, the following conclusions about resin-based splint materials can be drawn:

(1) The building orientation of additively manufactured materials has a significant effect on wear behavior and water sorption.
(2) The cleaning procedure after printing does not have a significant influence on either of the tested parameters.
(3) The type of post-polymerization does not have a significant influence on either of the tested parameters.
(4) The storage of the samples has a significant influence on the wear behavior.
(5) The water sorption is different between various additively manufactured materials.

## Figures and Tables

**Table 1:** Study design, materials and devices.

	Abbr. Text	Device/Material Protocol	Manufacturer LOT
Printer		P30+ (digital light processing)	Straumann, Basel, Switzerland
Orientation	0°		
	45°		
	90°		
Cleaning	AUTO	P Wash (isopropanol): pre-cleaning 3:10 min, cleaning 2:20 min, drying 1:30 min	Straumann, Basel, Switzerland
	MAN	Pre-/Main-Clean (isopropanol): pre-cleaning 3:00 min, ultrasonic: 2:00 min, air-drying: 1:00 min	VOCO, Cuxhaven, Germany
Post-polymerization	LED	P Cure: LED, 10 min, vacuum, UV–A: 400–315 nm; UV-B: 315–280 nm, heating	Straumann, Basel, Switzerland
	XEN	Otoflash G171: 2 × 2000 Xenon flashes, 280–700 nm, maximum between 400 and 500 nm	NK-OPTIK, Baierbrunn, Germany
Materials	M1	Luxaprint OrthoPlus: >90% bisphenol A dimethacrylate, 385/405 nm, flexural strength ≥ 70 MPa, flexural modulus ≥ 1 GPa, Shore D ≥ 60	DMG, Hamburg, Germany LOT 218479
	M2	V-Print Splint: acrylate, Bis-EMA, TEGDMA, hydroxypropyl methacrylate, butylated hydroxytoluene, diphenyl(2,4,6-trimethylbenzoyl) phosphine oxide, 385 nm, flexural strength 75 MPa, flexural modulus ≥ 2.1 GPa, sorption 27.7 μg/mm^3^, solubility < 0.1 μg/mm^3^	VOCO, Cuxhaven, Germany LOT 2023138

**Table 2:** Wear—Intermediate subject effects (significance of α = 0.01; R^2^ = 0.090; gray: significant effects).

	F	*p*-Value
aging	10.483	0.001
position	7.302	0.001
material	0.058	0.810
cleaning	0.007	0.933
polymerization	1.406	0.237
aging ∗ position	1.071	0.344
aging ∗ material	0.139	0.710
aging ∗ cleaning	0.011	0.916
aging ∗ polymerization	0.917	0.339
position ∗ material	1.696	0.185
position ∗ cleaning	2.638	0.073
position ∗ polymerization	1.010	0.365
material ∗ cleaning	0.692	0.406
material ∗ polymerization	0.120	0.729
cleaning ∗ polymerization	0.047	0.829
aging ∗ position ∗ material	2.687	0.070
aging ∗ position ∗ cleaning	1.711	0.182
aging ∗ position ∗ polymerization	1.174	0.310
aging ∗ material ∗ cleaning	0.456	0.500
aging ∗ material ∗ polymerization	1.442	0.231
aging ∗ cleaning ∗ polymerization	0.587	0.444
position ∗ material ∗ cleaning	4.583	0.011
position ∗ material ∗ polymerization	1.426	0.242
position ∗ cleaning ∗ polymerization	2.348	0.097
material ∗ cleaning ∗ polymerization	2.006	0.158
aging ∗ position ∗ material ∗ cleaning	2.178	0.115
aging ∗ position ∗ material ∗ polymerization	0.497	0.609
aging ∗ position ∗ cleaning ∗ polymerization	0.959	0.384
aging ∗ material ∗ cleaning ∗ polymerization	2.766	0.097
position ∗ material ∗ cleaning ∗ polymerization	0.457	0.634
aging ∗ position ∗ material ∗ cleaning ∗ polymerization	0.186	0.830

**Table 3:** Water sorption (Wsp)—Intermediate subject effects (significance of α = 0.01; R^2^ = 0.916; gray: significant effects).

	F	*p*-Value
position	23.273	<0.001
material	988.122	<0.001
cleaning	0.049	0.826
polymerization	0.907	0.343
position ∗ material	74.666	<0.001
position ∗ cleaning	13.298	<0.001
position ∗ polymerization	0.179	0.836
material ∗ cleaning	32.759	<0.001
material ∗ polymerization	1.245	0.267
cleaning ∗ polymerization	3.950	0.050
position ∗ material ∗ cleaning	13.732	<0.001
position ∗ material ∗ polymerization	3.657	0.030
position ∗ cleaning ∗ polymerization	1.974	0.145
material ∗ cleaning ∗ polymerization	0.230	0.632
position ∗ material ∗ cleaning ∗ polymerization	2.228	0.113

## Data Availability

The data presented in this study are available on request from the corresponding author due to privacy.
